# The Training of the Brain

**Published:** 1897-01-23

**Authors:** 


### The Hospital

A Journal of

The Medical Sciences and Hospital Administration,

[Vol. XXI.—No. 539.] [Jan. 23, 1897.

### The Training of the Brain.

America, almost more than England, recognises that no such thing as finality has been or can be reached in human effort and human achievement. The American medical and general writer and reader perceives this quite as clearly, and acts upon it with as alert a decisiveness as the American politician or man of business. The hand, we all understand, must be highly trained in an age when technical things must be done with technical capacity, or they will find no place in the world's markets. But if the hand in a technical age, much more the brain in an age when clear, intelligent, comprehensive, and decisive mental action is the first and most indispensable of all the conditions of immediate and permanent success. The competent brain may do much with half-competent hands, but the competent hand can do nothing great with a half-competent brain. There has just been published by Messrs. Macmillan and Co., in London and New York, a little volume written by Mr. N. P. Halleck, M.A., on the "Education of the Central Nervous System." Now all education, properly so-called, is education of the central nervous system, even when it is primarily education of the eye or the ear, the hand or the foot.

Has then Mr. Halleck, has anybody, any method of really making the human brain a more competent, reasoning, volitional, and directing instrument than it has hitherto been? And if Mr. Halleck has anything new to teach us about the training of the central nervous system, what, in a word, is it? As a matter of fact, he has nothing which is strictly new; but he calls the attention of the world to certain ideas in mental training which are "true" ideas; and it is much more important that ideas be true than that they be new. He insists, in short, that the central nervous system must be trained through the senses; and that some of the senses are more efficient as training mediums for certain purposes than others. Perhaps, in strict philosophy, that could with difficulty be proved; but the principles of action laid down by Mr. Halleck, both physiologists and general educationists will unhesitatingly agree with.

The ear and the eye are both "senses"; but the ear, we are told, is sometimes a better training medium than the eye. For example, a certain class of boys were told to read "As You Like It" for themselves, and were examined upon it afterwards by their teacher. They then had the play read aloud to them by the master, and were again examined upon it. It was found that they could remember, repeat, and give a far more intelligent account of the play after hearing it read aloud than after looking at the printed page and reading it for themselves. Hence it is concluded that the ear is a better sensory interpreter of Shakespeare than the eye. That, however, is hardly philosophical. It was not the boys' ears which interpreted "As You Like It" in that case, but the inflections of the master's voice, and the gestures of his body with the expressions of his face. If the play had been read aloud by one of the dullest boys in the class it is probable that the central nervous systems of the other boys would have received very much the same impressions through the ear as they did through the eye when each boy read the play for himself.

Is there then nothing in the principle of educating the central nervous system by sensory means? So far from there being nothing, there is everything in it. The thing is to use the right sense for the right purpose. As a general rule one would say that the eye is the prince of the senses, much more than the ear. Mr. Halleck, of course, insists, as we are now insisting, that every sense must be used for the training of the central nervous system, but each one to its fullest teaching capacity and in its own sphere. If we want to give the central nervous system the completest education in taste we must, we are told, blindfold the eyes and stuff up the nostrils. The central nervous system, without aid from the eyes or the nose, will then be called upon, by the agency of taste alone, to distinguish between roast goose, duck, and quail, or between strawberry, grape, and pineapple, as the convenience or the pocket of the schoolmaster may from time to time determine.

The main object in all education of the central nervous system we conceive to be this, to make the clearest, truest, most precise and most lasting impression upon it which can possibly be made; and for this purpose the best means is to act through the particular sense which is most likely to produce that impression. Sometimes to "see a thing for oneself" is the most effectual method of producing a true, deep, and lasting impression upon the central nervous system. At other times, the touching, taking up, and handling of a thing is more effective; in other cases, again, the hearing, or the tasting, or the smelling, or the feeling of pain, may give the truest and deepest impression. But, whatever makes the consciousness realise actualities most fully, is above all things the most effectual instrument for the education of the central nervous system.

There is, we repeat, nothing very new in all this. For all great teachers and reformers, in all ages, from Moses and Confucius downwards, have devoted their highest powers to the producing of deep and lasting impressions upon the "central nervous system." Only, until recent years, they did not call the central nervous system by that name, nor did they dream of its existence. We do what they did, and for the same ends; but, thanks to physiology, we know a little better what we are about, and so we can sometimes adapt means to ends with more intelligence and, therefore, with more just confidence. Everybody of any capacity and experience will wish well to all honest and intelligent efforts for the better training of the "central nervous system."

